# Benchmarking Orthogroup Inference Accuracy: Revisiting Orthobench

**DOI:** 10.1093/gbe/evaa211

**Published:** 2020-10-06

**Authors:** David M Emms, Steven Kelly

**Affiliations:** Department of Plant Sciences, University of Oxford, United Kingdom

**Keywords:** orthogroup, orthology, benchmark

## Abstract

Orthobench is the standard benchmark to assess the accuracy of orthogroup inference methods. It contains 70 expert-curated reference orthogroups (RefOGs) that span the Bilateria and cover a range of different challenges for orthogroup inference. Here, we leveraged improvements in tree inference algorithms and computational resources to reinterrogate these RefOGs and carry out an extensive phylogenetic delineation of their composition. This phylogenetic revision altered the membership of 31 of the 70 RefOGs, with 24 subject to extensive revision and 7 that required minor changes. We further used these revised and updated RefOGs to provide an assessment of the orthogroup inference accuracy of widely used orthogroup inference methods. Finally, we provide an open-source benchmarking suite to support the future development and use of the Orthobench benchmark.

**Significance**

Orthogroup inference forms the foundation of comparative genomic analysis. Benchmarks to evaluate performance are essential to enable these methods to be compared and stimulate further method development. Here, we present an update to the Orthobench benchmark database and provide a comparative performance evaluation of commonly used orthogroup inference methods.

## Introduction

Determining the phylogenetic relationships between genes is fundamental to comparative biological research. Although pairwise comparisons between species typically leverage the use of orthologs (i.e., genes in those species that evolved from a single ancestral gene by speciation), comparisons across multiple species require the use of orthogroups (i.e., the complete set of genes descended from a single ancestral gene in the last common ancestor of the species being analyzed). Furthermore, phylogenetic analysis of orthogroups provides the basis for our understanding of the diversity and evolutionary history of life on earth. Given the fundamental utility of orthogroups in comparative biological research, several automated methods have been developed to identify them from raw sequence data. Widely used methods include OrthoMCL (Li et al. 2003), OMA (Train et al. 2017), Hieranoid (Kaduk and Sonnhammer 2017), OrthoFinder (Emms and Kelly 2015), and SonicParanoid (Cosentino and Iwasaki 2019). Each of these methods adopts different approaches to the challenges introduced by gene duplication and loss, unequal species sampling, and differential rates of sequence evolution.

Given the methodological differences between inference methods it is important to have accurate benchmarking tools to enable their assessment. The benchmarking tests and methodologies used by the Quest for Orthologs Consortium provide an critical resource for enabling the evaluation of ortholog inference accuracy (Gabaldon et al. 2009; Dessimoz et al. 2012; Sonnhammer et al. 2014; Altenhoff et al. 2016, 2020; Forslund et al. 2018; Glover et al. 2019). However, benchmarking tests of orthogroup inference accuracy are limited. The original Orthobench study (Trachana et al. 2011) was an exemplary contribution in this endeavor. In this study, the authors provided 70 expert-curated Bilateria-level orthogroups, which were termed reference orthogroups (RefOGs). Each of these RefOGs was intended to comprise the complete set of genes that are descended from a single copy gene in the most recent common ancestor of the Bilateria. The 70 RefOGs exemplified a range of biological and technical factors that challenge orthogroup inference methods. The authors assembled these orthogroups through expert analysis of rooted gene trees inferred from multiple sequence alignments (MSAs). This benchmark database has acted as a gold standard against which orthogroup inference methods have been tested for nearly a decade.

Over the last decade, tools for MSA and tree inference have improved considerably (Sievers and Higgins 2020). Of particular note are the improvements that have been made in phylogenetic tree inference. These include automated testing for the best fitting model of sequence evolution, new topology search strategies, higher-computational efficiency, and improved parallelization (Stamatakis 2014; Nguyen et al. 2015; Kalyaanamoorthy et al. 2017). These improvements, coupled with vast increases in computational power, make feasible the inference of multiple, larger gene trees using better-fitting substitution models. Unlike a decade ago, gene trees of hundreds of genes can be inferred with ease, allowing exploratory testing of phylogenetic hypotheses. This removes the need for tight inclusion thresholds which can exclude both true-positive orthogroup members and important context from the wider gene family necessary for accurate placement of the root of the gene tree under consideration. Thus, it is timely to re-evaluate the Orthobench benchmark database and reassess membership of its 70 RefOGs, aided by the technological advancements since the original study.

Here, we utilized up-to-date bioinformatic methods to conduct an ab initio search, alignment, and phylogenetic evaluation of the 70 RefOGs from the original Orthobench database (Trachana et al. 2011). Using this approach, we revised the membership of 31 of the 70 RefOGs (44%). Although seven of the RefOGs required minor revision to orthogroup membership, 24 of the RefOGs required major revision that altered the phylogenetic extent of the genes in the orthogroup. To facilitate future use of this resource, we provide the complete revised benchmark database and testing suite to enable orthogroup inference methods to be evaluated on this benchmark. This testing suite also includes the input proteome data sets and analysis scripts to compute the benchmark scores. Finally, to ensure reproducibility and promote further improvements to the benchmarks, the complete working data set and a summary of the evidence used to determine each updated RefOG is available from https://github.com/davidemms/Open_Orthobench.

## Results

### Inference of Gene Trees of RefOGs in the Context of Their Wider Gene Families

The first step in the assessment and potential revision of the RefOGs from Orthobench required the identification and retrieval of sets of genes from the 12 species used in the original study (Trachana et al. 2011). Given that there had been updates to many of the genomes’ annotations since the publication of the study, the latest versions of the proteomes for the original 12 species were downloaded and are provided (Zenodo research archive https://doi.org/10.5281/zenodo.4015193 and https://github.com/davidemms/Open_Orthobench). The proteomes for an additional three outgroup and two ingroup species were also downloaded for use in this analysis. These additional species were used to provide additional evidence during the manual curation phase of the RefOG assessment but, for consistency, do not form part of the final benchmarks.

Sequence similarity searches were conducted using HMMER (Mistry et al. 2013) with the 70 hidden Markov models (HMMs) prepared in the original study (Trachana et al. 2011) used as queries. For each RefOG, the set of sequences that were used for subsequent alignments and tree inference were selected directly from these HMM search results using a variable *e*-value threshold. Liberal *e*-value inclusion thresholds were used to ensure that all putative members of each orthogroup were recovered at the expense of including large numbers of false positive (FP) genes in the initial gene tree (i.e., genes that belong to the same gene family but not the target RefOG). This was done as such FP genes are best identified and discarded on the basis of evidence provided in a gene tree, instead of on the basis of HMMER *e*-values. As a starting point, the least significant *e*-value for a sequence in the RefOG tree from the original study was determined and a new *e*-value threshold was chosen such that there would be three times as many genes included in the gathered set than would be have been included using the *e*-value implied by the tree from the original study. This strategy was supported by a subsequent analysis of the final, phylogenetically determined RefOGs. It showed that a median of 1.8 times as many genes achieved HMMER *e*-values better than the worst scoring true member of the RefOG as there were true members of the RefOG (supplementary fig. S1, Supplementary Material online).

Each gathered set of genes was subject to MSA using the MAFFT L-INS-i algorithm (Katoh and Standley 2013), alignment trimming using TrimAL (Capella-Gutierrez et al. 2009), and phylogenetic inference using IQ-TREE (Nguyen et al. 2015) with the best fitting model of sequence evolution inferred directly from each alignment (see Materials and Methods). This set of gene trees informed the subsequent phylogenetic analyses.

### Delineation of Bilateria-Level Orthogroups from within Large Gene Trees

**Fig. 1.**
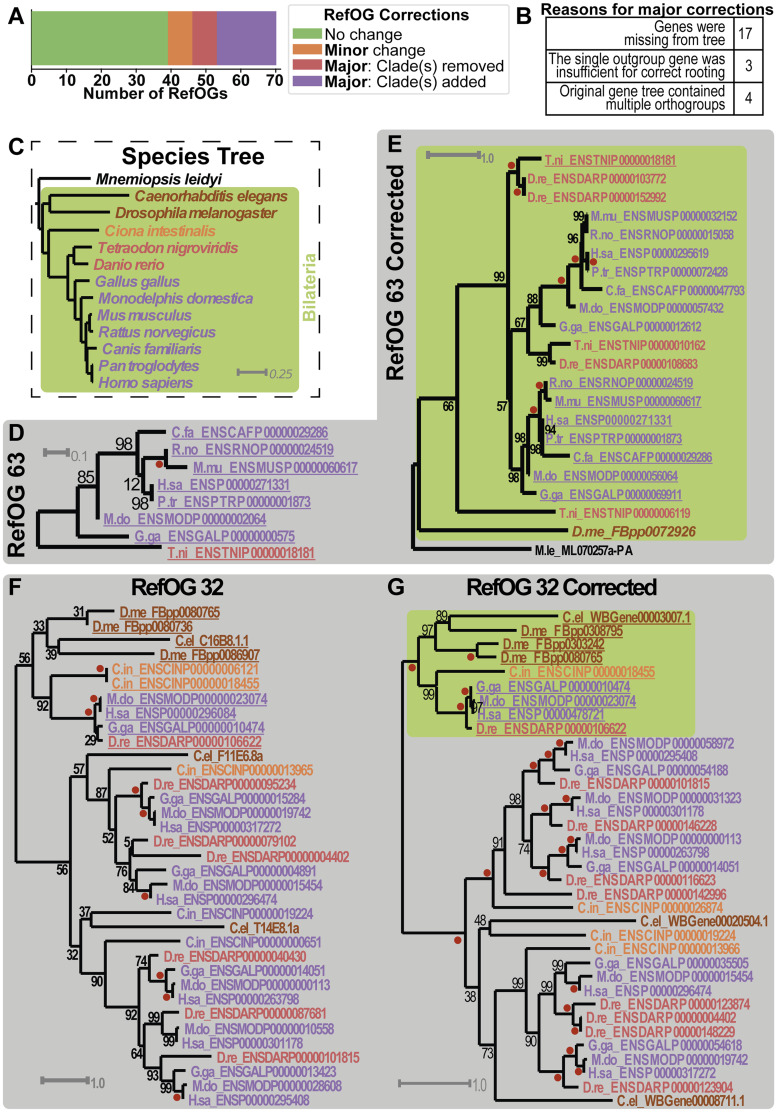
Evaluation and revision of RefOGs from Orthobench. (*A*) Summary of the corrections made to the RefOG data set. (*B*) Reasons for major corrections to RefOGs from the previous study. (*C*) The species tree. Green shaded area shows the 12 Bilaterian species for which the Bilateria-level orthogroups (RefOGs) were defined. One outgroup species, which appears in the gene trees in the figure, is also shown. (*D*) Example of a major improvement for which clades had been missing from the original RefOG tree: RefOG 63 gene tree as determined in the original study. (*E*) Gene tree from this study showing the corrected RefOG 63 orthogroup shaded green. Phylogenetic analysis revealed that the original RefOG32 comprises two separate orthogroups that diverged at a gene duplication even preceding the divergence of the vertebrates. (*F*) Example of a major improvement for which extra clades of genes had been included in the original RefOG: RefOG 32 gene tree as determined in the original study. (*G*) Gene tree from this study showing the corrected RefOG 32 orthogroup. Phylogenetic analysis revealed that these genes diverged from the remaining genes in the tree at a gene duplication event predating the divergence of the Deuterostomes and Protostomes. Gene trees show previously identified orthogroup containing the newly delimited orthogroup from this study (green shaded clade). Genes/species are colored according to species. Corresponding genes identified as members of the orthogroup in both studies are underlined (including when identifiers have been updated). Red dot = 100% bootstrap support.

The newly inferred gene trees were examined side-by-side with the trees from the original Orthobench study. In each case, the Bilateria-level orthogroup was determined and a comprehensive discussion of the evidence considered was recorded (see supplementary text S1, Supplementary Material online, and https://github.com/davidemms/Open_Orthobench). Overall, 56% of RefOGs (*n* = 39) from the previous study were confirmed with no changes to their extent. A further 10% (*n* = 7) required only minor revision, affecting multiple genes. The remaining 34% of RefOGs (*n* = 24) required major revision affecting the phylogenetic extent of the species with representatives in the RefOG (fig. 1*A* and supplementary table S1, Supplementary Material online).

The most common reason for major revision of a RefOG (i.e., affecting the phylogenetic extent) was that phylogenetically relevant genes were missing from the gene tree inferred in the original study. The revised phylogenetic trees support the inclusion of these genes and thus no evidence can be discerned for their original exclusion (fig. 1*B*–*E* and supplementary table S1, Supplementary Material online). It is possible that these genes did not meet the *e*-value threshold used for the RefOG in the original study, but the data were not available to assess this. The lenient initial threshold combined with the use of the gene trees to assess RefOG extent in the present study was designed to prevent the occurrence of such missing genes.

In addition to exclusion of clades of genes, there were also three cases of overinclusion of clades of genes (supplementary table S1, Supplementary Material online). This overinclusion was likely caused by misinterpretation of gene duplication events that occurred prior to the divergence of the Bilateria. These gene duplication events should have resulted in the identification of two Bilaterian orthogroups rather than one. This error may have occurred as there was only a single outgroup gene in the original trees and thus there was insufficient evidence to unambiguously root the tree correctly with respect to the gene duplication event (supplementary fig. S2, Supplementary Material online). In four further cases, there was ambiguous or unambiguous evidence in the original tree that it contained multiple orthogroups, and further phylogenetic investigation confirmed this (fig. 1*F* and *G* and supplementary table S1, Supplementary Material online). A further 10% of RefOGs required minor revision, involving the addition or removal of single genes within already correctly identified clades. Thus, overall 44% of RefOGs required revision.

### Evaluation of Orthogroup Inference Methods Using the Updated Benchmarks

**Fig. 2.**
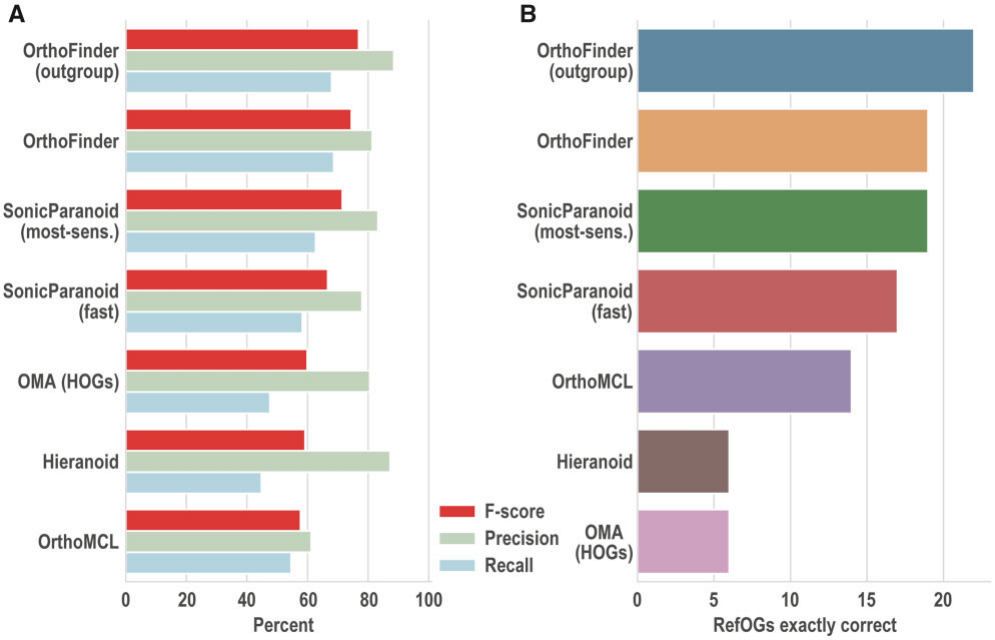
The benchmark results for the methods tested. (*A*) Precision, recall, and *F*-score. (*B*) Number of orthogroups predicted exactly, with no extra or missing genes.

As the revised RefOGs were computed through manual gathering of genes and phylogenetic trees, they are methodologically independent of all orthogroup inference methods. Thus, they serve as an ideal benchmark data set on which to compare the performance of orthogroup inference methods. To facilitate the use of these RefOGs as an orthogroup benchmark, a complete benchmarking suite was prepared that included the 12 input proteomes, the revised RefOGs, and a script for calculating the benchmarks for a set of inferred orthogroups. A set of commonly used orthogroup inference methods comprising OrthoFinder (Emms and Kelly 2019), SonicParanoid (Cosentino and Iwasaki 2019), OrthoMCL (Li et al. 2003), Hieranoid (Kaduk and Sonnhammer 2017), and OMA HOGs (Train et al. 2017) were tested against the benchmarks. Most methods had high precision (80 ± 8%) but lower recall (fig. 2*A* and supplementary table S2, Supplementary Material online). The biggest impact on recall for all methods was incorrectly splitting orthogroups rather than from individual missing genes (supplementary fig. S3, Supplementary Material online). The OrthoFinder (with outgroup) achieved the highest overall accuracy (*F*-score) and precision. OrthoFinder (default) achieved the highest recall and second highest *F*-score. The ordering of the methods according to the number of RefOGs inferred exactly (no missing or extra genes) was similar to the ordering by *F*-score (fig. 2*B*). In agreement with the observed differences in accuracy, and consistent with the differences in methodological approach, the orthogroups predicted by the methods also showed differences in the orthogroup assignment of all genes as measured by the adjusted rand score (supplementary fig. S4, Supplementary Material online).

### Inference Accuracy on Biologically and Technically Challenging Orthogroups

**Fig. 3.**
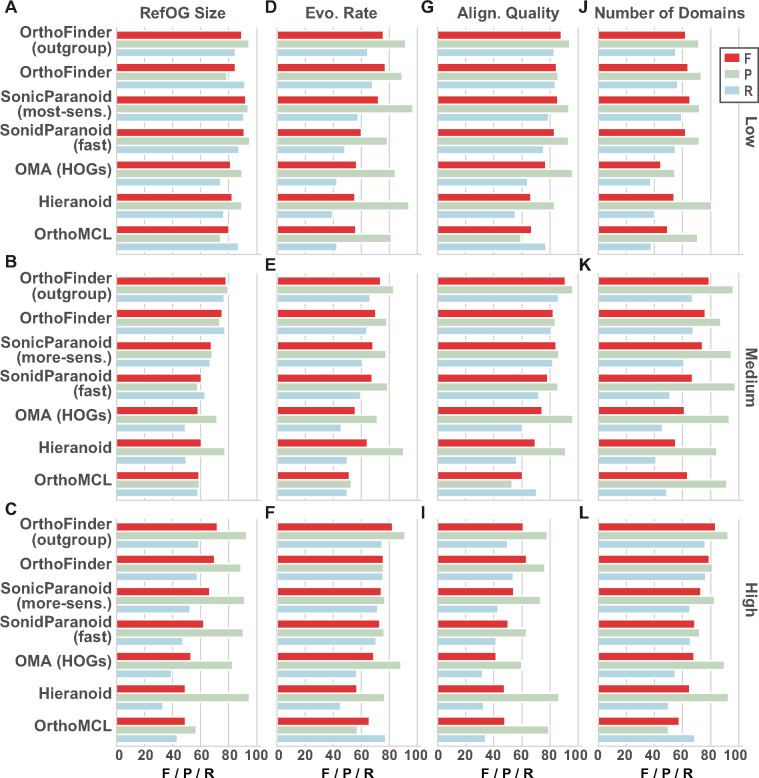
Breakdown of the precision (P), recall (R), and *F*-score (F) of the methods under different levels of technical challenges to orthogroup inference. (*A*–*C*) RefOG size, *N*. (*A*) Low, *N* < 15; (*B*) medium, 15 ≤ *N* < 31; and (*C*) high, *N* ≥ 31. (*D*–*F*) Evolutionary rate measured my mean sequence identity, *I*. (*D*) Low evolutionary rate, *I* > 73.8%; (*E*) medium, 62.4% < *I* ≤ 73.8%; and (*F*) high, *I* ≤ 62.4%. (*G*–*I*) Alignment quality, *Q* = norMD. (*G*) Low, *Q* < 0.88; (*H*) medium, 0.88 ≤ *Q* ≤ 1.15; and (*I*) high, *Q* > 1.15. (*J*–*L*) Number of domains, *D*. (*J*) Low, *D* = 1; (*K*) medium, 2 ≤ *D* ≤ 3; and (*L*) high, *D* > 3.

In the original Orthobench study, a number of challenges to orthogroup inference accuracy were identified: gene duplications/loss, rate of sequence evolution, domain architecture, and alignment quality (Trachana et al. 2011). To assess the impact each of these factors has on orthogroup inference accuracy, each RefOG was categorized as in the lowest, medium, or highest third of the 70 RefOGs for each factor and the accuracy of each method was assessed on each of these partitions of the RefOGs (see Materials and Methods). Each of these factors was found to affect the accuracy of all methods (fig. 3). All methods had high *F*-score for the smallest RefOGs. However, (recall and therefore *F*-score) was reduced for the largest RefOGs (fig. 3*A*–*C*). The rate of sequence evolution had a lesser effect on orthogroup inference accuracy (fig. 3*D*–*F*). Poor MSA quality was associated with lower recall and therefore *F*-score for all methods (Figs. 2 and 3*G*–*I*), although it should be noted that the methods themselves do not use MSAs. RefOGs with the smallest number of domains presented the greatest challenge to orthogroup inference, with each method achieving lower recall and *F*-score. Overall, challenges to orthogroup inference typically resulted in poor recall (genes missing from orthogroups) with higher precision for the few genes assigned to the correct orthogroups (fig. 3).

### An Open-Source Benchmark to Encourage Future Improvements

An archive of the complete set of data used to determine the members of each orthogroup is provided as Supplementary Material online (Zenodo research archive https://doi.org/10.5281/zenodo.4015193). A GitHub repository has also been created to allow the community to rapidly identify if any further improvements should be made to the benchmarks and to allow the benchmarks to be updated accordingly: https://github.com/davidemms/Open_Orthobench. For each RefOG, the following data are provided: the HMMER search results against each of the proteomes; the FASTA file of the selected sequences for subsequent tree inference; the MSA of these selected sequences; the rooted gene tree inferred from this alignment; and a commentary outlining the evidence considered in determining the extent of the orthogroup. The inclusion of the evidence considered allows the reasoning used in each case to be checked. It also allows any future reanalysis to take into account all the evidence already collected, and to identify what new evidence is available to support any further correction. The GitHub repository also included the input sequence files and a benchmarking script to allow any new orthogroup inference method to be tested using consistent input data and methodology.

## Discussion and Conclusions

Orthogroup inference forms the foundation of comparative genomic analysis in the postgenome era. Methods for orthogroup inference are well developed; however, resources for orthogroup inference benchmarking are limited. The Orthobench (Trachana et al. 2011) orthogroup inference benchmark has been the gold standard for benchmarking the accuracy of orthogroup inference methods for nearly a decade. Among the 70 Bilaterian orthogroups, it contains 35 which were selected as particularly challenging for phylogenetic analysis and orthogroup inference. In this study, we revealed that 39% of orthogroups in the Orthobench database were incorrect and needed revision. In providing these revisions, we have generated a new open-source repository for the benchmarks, including all input datafiles and assessment scripts. This revised benchmark database provides more accurate assessment of orthogroup inference methods and will guide future methodological improvements.

We evaluated widely used orthogroup inference methods on the revised benchmarks. This illustrated the wide range in performance characteristics exhibited by these methods. Although some methods did better than others on different components of accuracy (such as precision and recall), all were challenged by the benchmarks. The largest differences were between the recall achieved by different methods, with less accurate methods failing to place genes together in the same orthogroups. Low recall was also the most common problem observed for biologically or technical challenging orthogroups. Overall, the best performing methods were ∼77% accurate and the most accurate method only got 23 of the 70 RefOGs exactly correct. Although these RefOGs were deliberately chosen to test the limits of orthogroup inference, this analysis reveals that there is still considerable scope for methodological improvement to tackle the most challenging gene families.

As these methods form the foundation of thousands of comparative genomic analyses annually, such methodological improvements have the potential to drive improvements across the breath of comparative genomic research. To aid future benchmarking and accelerate any future efforts to improve the benchmarks and methods further, the complete, open-source benchmarking suite has been made available on https://github.com/davidemms/Open_Orthobench.

## Materials and Methods

### Data Availability and Open-Source Benchmarking Repository

The input proteomes for each of the 12 species in the original Orthobench study (Trachana et al. 2011), as well as for the three outgroup species: *Mnemiopsis leidyii*, *Trichoplax adhaerens*, and *Nematostella vectensis* and two additional ingroup species: *Branchiostoma lanceolatum* and *Schistosoma mansoni* (diverging early within the Deuterostomes and Protostomes, respectively), were downloaded from Ensembl (Cunningham et al. 2019) in March 2020. Version numbers for all proteomes are provided in supplementary table S3, Supplementary Material online. All input proteomes, HMMER search results, sequence files, alignments, trees, and commentaries are provided as a supplementary data archive, Supplementary Material online (Zenodo research archive https://doi.org/10.5281/zenodo.4015193). These data are also provided as an open-source GitHub repository (https://github.com/davidemms/Open_Orthobench) so that if any further improvements are identified by the community, the benchmarks can be updated accordingly. The repository also contains the script for benchmarking a set of predicted orthogroups. For each RefOG, a file has been provided that details the analysis performed and the evidence that has been weighed in determining the members of each Bilateria-level orthogroup. Users of this resource should also cite the original study (Trachana et al. 2011).

### Reference Proteomes for Use in RefOG Construction

Orthogroups were constructed using a single representative gene model for each gene locus. To facilitate this, a FASTA file was prepared for each species containing the protein sequence corresponding to the longest transcript variant for each gene locus. Most orthogroup inference methods assume either implicitly or explicitly that only a single variant for each gene is included in the input files under consideration. However, both the 12 complete proteomes and the 12 “longest transcript variant” proteomes are provided so that either can be used by an orthogroup inference method to test against the benchmarks. Additionally, methods that make use of outgroup species to help delineate orthogroups can use the species used in this study or any alternate species judged to be suitable for the orthogroup inference method.

### Construction of Extended Gene Sets for Gene Tree Inference

The HMM from each of the RefOGs generated in the original Orthobench study was searched against the longest transcript variant proteomes using HMMER (Mistry et al. 2013) with the command, “hmmsearch -E 0.001 --max.” As a starting point, the highest *e*-value for a sequence in the RefOG tree from the original study was determined and a new *e*-value threshold was chosen such that there would be three times as many genes included in the gathered set than would be included using the *e*-value implied by the tree from the original study. BLAST (Camacho et al. 2009) and DIAMOND (Buchfink et al. 2015) were also employed for additional searches as detailed in the evidence files associated with each RefOG. The *e*-value threshold, gathered set size, and final RefOG size are provided in supplementary table S4, Supplementary Material online.

### Inference and Editing of MSAs

Protein sequences were aligned using MAFFT L-INS-i (Katoh and Standley 2013) with default parameters and trimmed using TrimAl using the options “-gt 0.5” to remove columns from the MSA that contained more than 50% gaps. The median percentage of gap characters for the columns removed in this way was 93% (supplementary table S4, Supplementary Material online). AliView (Larsson 2014) was used to examine MSAs. Short MSAs were only trimmed to remove columns with more than 75% gaps, or not at all. This requirement was judged on a case-by-case basis. The alignment lengths and number of trimmed columns for each RefOG are provided in supplementary table S4, Supplementary Material online.

### Inference of Gene Trees

In all cases, phylogenetic trees were inferred using IQ-TREE 1.6.11 using the options “-m TEST” to identify the best fitting model in each case and “-bb 1000” to perform a bootstrap analysis. The model parameters for each RefOG are provided in supplementary table S4, Supplementary Material online. Parallelization of multiple commands was performed using GNU Parallel (Tange 2011). Gene trees were analyzed using Dendroscope (Huson and Scornavacca 2012) and the ETE library (Huerta-Cepas et al. 2016). The complete analysis of each RefOG is detailed in supplementary text S1, Supplementary Material online.

### Comparative Evaluation of Orthogroup Inference Methods

Published methods that had been submitted to the latest Quest for Orthologs benchmarks server (Altenhoff et al. 2016, 2020) and which inferred orthogroups were selected for benchmarking. Where the methods presented multiple options, both the fastest and the most accurate options were tested. The methods tested were Hieranoid v2 (Kaduk and Sonnhammer 2017), OMA (HOGs) v2.4.1 (Train et al. 2017), OrthoFinder v2.4.0 (Emms and Kelly 2019), OrthoFinder v2.4.0 (outgroup), OrthoMCL v2.0.9 (Li et al. 2003), SonicParanoid v1.3.0 (fast) (Cosentino and Iwasaki 2019), and SonicParanoid v1.3.0 (most-sensitive). Each orthogroup inference method was run on the 12 longest transcript variant proteomes (plus three outgroup proteomes for OrthoFinder [outgroup]). Hieranoid, OMA, and OrthoFinder (outgroup) were additionally provided with the rooted species tree. Programs were run with default options other than those required for the variations described above. For those cases where they were required, the command line options, configuration files, and species tree used are provided in Supplementary Material online as are the files of orthogroups predicted by each method.

Every pair of genes in the same predicted orthogroup that was also in the same RefOG was counted as true positives (TPs). Likewise, pairs of genes in the same predicted orthogroup but not in the same RefOG were counted as FPs and vice versa for false negatives (FNs). The TP, FP, and FN were divided by (*n* − 1), for each RefOG, where *n* is the number of genes in the RefOG, to normalize for RefOG size. Without this normalization, the overall scores would be biased toward the larger RefOGs. Note that a single gene missing from a RefOG results in (*n* − 1) fewer/more TPs/FNs, thus (*n* − 1) is the correct normalization for RefOG size rather than the number of pairs, *n*(*n* − 1)/2, because such a score would bias the benchmarks toward the smallest RefOGs. The normalized TP, FP, and FN were summed over the RefOGs and the precision, recall, and *F*-score were calculated from these. A small number of genes were identified as members of the RefOGs but only with low certainty (see Supplementary Material online), these genes were discounted when calculating the number of TP, FP, and FN pairs. Including also these low certainty genes had little effect on the relative accuracy of the methods (supplementary fig. S5, Supplementary Material online).

### Technical Challenges for Orthogroup Inference

To evaluate the impact of different biological or technical challenges to orthogroup inference, each RefOG was classified based on the orthogroup size, rate of sequences evolution, alignment quality, and domain complexity, as for the original Orthobench study (Trachana et al. 2011) except for domain complexity, where a reproducible method was instead described in Trachana et al. (2014). Rate of evolution was evaluated using the mean sequence identity score, described as the “FamID” score in Muller et al. (2005). Alignment quality was evaluated based on the norMD score (Thompson et al. 2001) calculated using the NORMD tool version 1.3 (Muller et al. 2010). Domain complexity was evaluated by calculating the average number of protein domains for genes within the genes in each RefOG. The protein sequences were searched against the Pfam 31.0 database (El-Gebali et al. 2019) using HMMER 3.3 (Mistry et al. 2013). “Family-specific conserved domains” were identified as those domains present in 75% or more of genes within the RefOG. Domain complexity was classified based on the number of family-specific conserved domains within the RefOG. For each of the four classification, the RefOGs were split into terciles containing the lowest, middle, and highest third (or as close as possible for discrete data: RefOG size and number of domains) according to the measure. Each orthogroup inference method was then evaluated on each of these subsets using the precision, recall, and *F*-score described above.

## Supplementary Material

Supplementary data are available at *Genome Biology and Evolution* online.

## Supplementary Material

evaa211_Supplementary_Data
